# Supplementary material for: Purifying and balancing selection on embryonic semi-lethal haplotypes in a wild mammal
Source: Evol Lett. 2023 Oct 23;8(2):222–30. doi: 10.1093/evlett/qrad053 (PMC10959477; doi:10.1093/evlett/qrad053)
Supplement: qrad053_suppl_Supplementary_Material [file qrad053_suppl_supplementary_material.pdf]

Supplementary material

Purifying and balancing selection on embryonic semi-lethal haplotypes in a wild mammal.

Authors names and addresses:

Stoffel, M.A.[1*], Johnston, S.E.[1], Pilkington, J.G.[1], Pemberton, J.M[1]

[1]Institute of Evolutionary Biology, School of Biological Sciences, University of Edinburgh, Edinburgh, EH9 3FL, United Kingdom

Short running title:
Embryonic semi-lethal mutations in a wild mammal

* Corresponding author:
Martin A. Stoffel
Postal address: Institute of Ecology and Evolution, University of Edinburgh, Edinburgh, EH9 3FL, UK

E-mail: martin.stoffel@ed.ac.uk

Supplementary Figures

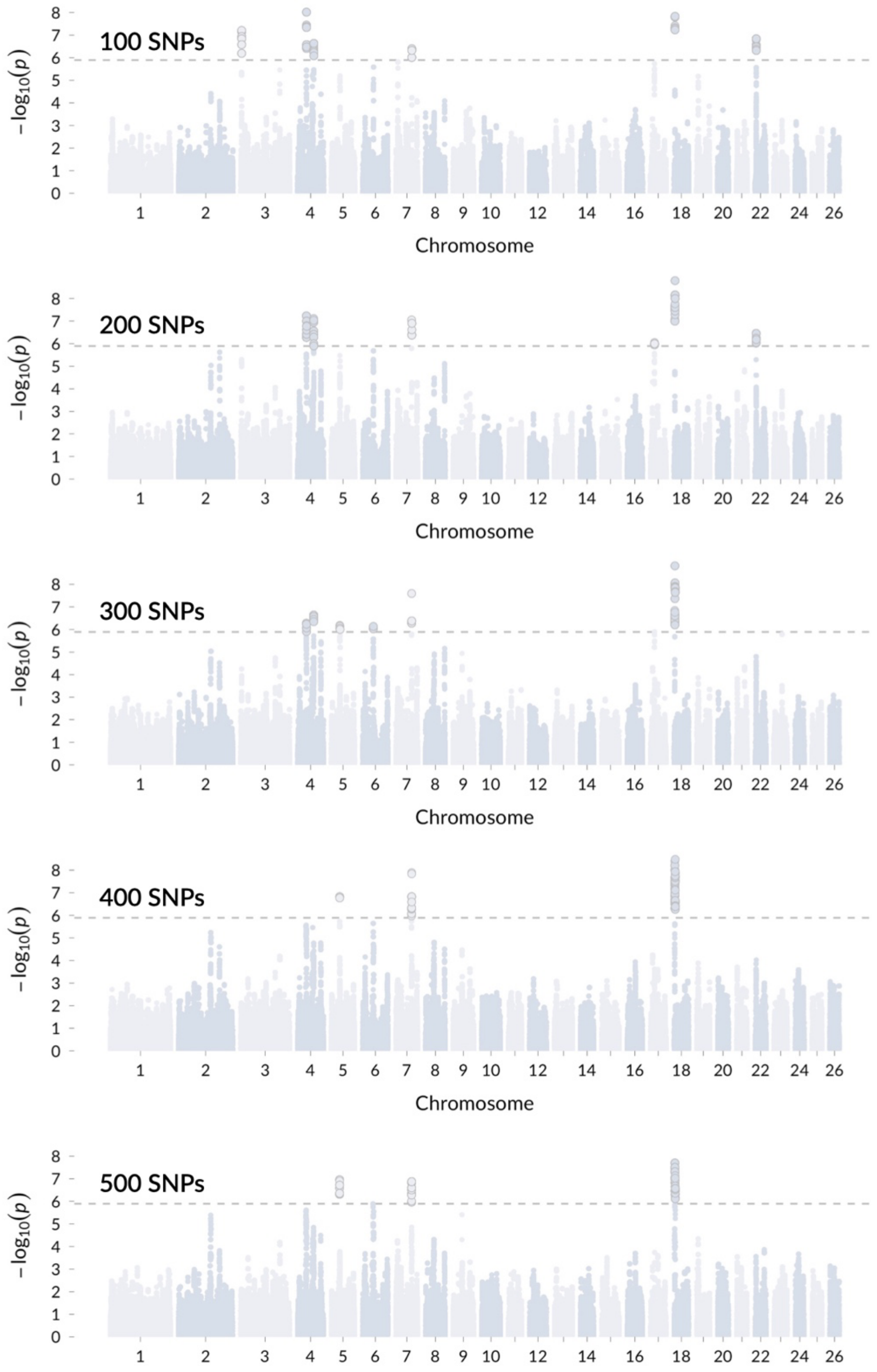

Supplementary Figure 1: Genome-wide scans for depleted haplotype homozygosity in offspring of carrier x carrier matings for different haplotype lengths (100-500SNPs).

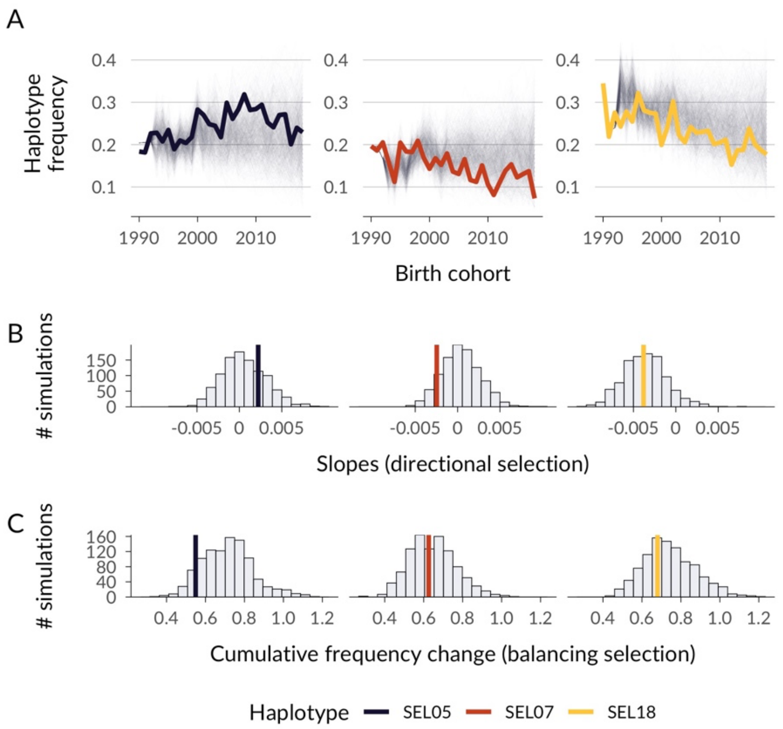

Supplementary Figure 2: Results of gene-dropping simulations with recombination. The genetic map length for each haplotype was determined using the interpolated high-density genetic map from Stoffel, Johnston, Pilkington, & Pemberton, (2021), resulting in map lengths of 2.1, 1.9, and 4.4 cM for SEL05, SEL07, and SEL18, respectively. Cross-overs were simulated on these haplotypes in 2.1%, 1.9%, and 4.4% of meioses, respectively. Panel B shows that this resulted in 74%, 10%, and 47% of slopes being steeper than observed empirically for SEL05, SEL07, and SEL18. Panel C shows that it also resulted in 10%, 46%, and 34% of simulations being more stable than SEL05, SEL07, and SEL18. For simplicity, we did not simulate situations where a focal haplotype re-emerges due to recombination, which is not unlikely given the relatively limited genetic diversity in the Soay population.

Supplementary Tables

Haplotype	p value (chi-square test)	# observed homozyous offspring	# expected homozygous offspring	# carrier x carrier matings	chromosome	first SNP / last SNP	start position / end position (bp)
AAGGACAGGGACAAAGGAGAGGAAAGAGGACAGAGAGAAAAGAGACGGAG<br>AAGGAGGGGCAGGAGCGAAACGGAACAGAAGGGAGAAGAAAAAGGAAAGA<br>GAAGCAAGAAGAGAGGAAAAGAGAGAGAGAGCAAAGAGGAGGAGGAACGA<br>GAGAAAAGAAAAACGAGACGGAGCACAGGGGGGAGAAGGCACGAAGGGAG<br>GGAGCCACGGGAAAAGAGAAGGGAAGAAAAGGAGCGGAAGAGGGGGAAGC<br>GAGCAGAGAGGAGGGAGAAGGGACGAGGAAAACAAGAACGAAGAGGAAGA<br>GAGGAGCAGGGGAGACAAAAGGGAGAACGAAACGAGAAGGGAAAGAGACG<br>CGGAAAAAAGAAAACGAGAAGAGGAGAAGGAAAGAAAAAAAAAAAAAAGG	3.29e-09	176	254.25	815	18	oar3_OAR18_4691643-<br>oar3_OAR18_7448353	4691643-<br>7448353
GACGAACAAAGGGAAAGAAGAAGAAAAAGAAAAGAGAGGAAGAGCGAGAG<br>GAGGAGAAAAGAGACAAGAGAGGAAAGGAGGGAGCGAAGGGAGAAAAAGA<br>GAGAGGAGGCAAAAAAGACGAGAAAGGAAAGGCGAGCGCAAAAAGGAGAA<br>GAGGAAGAGAGAGAGAGCAAAGAAAGAGAACCAGAGGAAGAGAGAAGACA<br>AGGAAGAAAGAAAAACAAAGGAAAAAAGAGAGGGCGAGGAGGGGGAGAAA<br>AGAAGAAAAAGGGAGACGAAAAGGAACAGGGAGAAACAAGAAGACAAAAA<br>CAAGAAGCGGAATGGAAAGAACGAAGCCGAAAACGAGAGACGAAGAGAAA<br>GGGAAAGGAGAAAACAAACAACACCCGAGCGAGAGAAAAGAGAACGAAGG	1.28e-08	56	105.75	382	7	oar3_OAR7_71164579-<br>oar3_OAR7_73326795	71164579-<br>73326795
AGCGGACAAAGGAGAAAGAGGAAAAAACAGAAAACGAAAGAGCGCAGAAC<br>GCAGAAAGAGGCCGGGCGGCGAAAAACGAGGGGGGGCGGGACGAAAGAAG<br>GAGGAAGGGCAAGCAGAGGCAAAAGAAGAGTGAGGAAAAAAGGAAAAGAA<br>AAGAGGACAAGAAAAAAAAGGAGAGGAAGGAGAAACAAAGAGGAGGGGAA<br>AGGGGAGGGAAGGAAAACTAGCAAAGAACAAAGAAGAGGAGGGAAAGGAA<br>AGAGGAGCACAGGGGGAGAGGAGGGAGAAGAAACGGGAAAGGGACAGAAA<br>GCGCACGAAAGCGGAGGGGAAAGAAAGGGACAAAGAAGGGGAGGGAGCAA<br>GGGAGAAGGAGAGGGAGAAAGAGGGAAGAAAAAGGGAGCGAAGAGAAAAA	1.49e-07	189	258.50	800	5	oar3_OAR5_37164925-<br>OAR3_212455420.1	37164925-<br>39808507

Supplementary Table 1: Putatively semi-lethal haplotypes. Shown are the top hits from a genome-scan for depleted haplotype homozygosity in Soay sheep, their chi square p-values, the number of observed and expected homozygous offspring and the overall number of mating pairs as well as the location of the haplotypes in the sheep Oar_v3.1 sheep genome assembly. Note that 'Haplotype' shows the alleles at consecutive SNPs on the SNP chip, and not the genome-sequence.

Term	Post.Mean	CI (2.5%)	CI (97.5%)	Info
Intercept	0.46 (1.584)	-0.367 (0.693)	1.31 (3.705)	
Population level/fixed effects				
SEL05 (1 copy)	0.275 (1.316)	0.015 (1.015)	0.539 (1.715)	categorical
SEL05 (2 copies)	0.089 (1.093)	-0.453 (0.636)	0.619 (1.857)	categorical
SEL07 (1 copy)	-0.236 (0.79)	-0.521 (0.594)	0.045 (1.046)	categorical
SEL07 (2 copies)	0.159 (1.172)	-0.783 (0.457)	1.106 (3.021)	categorical
SEL18 (1 copy)	-0.009 (0.991)	-0.265 (0.767)	0.243 (1.275)	categorical
SEL18 (2 copies)	-0.026 (0.974)	-0.557 (0.573)	0.51 (1.665)	categorical
F_{ROH}	-0.08 (0.923)	-0.205 (0.814)	0.044 (1.045)	z-transformed (x-mean(x))/sd(x)
Hindleg length	0.636 (1.89)	0.486 (1.625)	0.793 (2.21)	z-transformed (x-mean(x))/sd(x)
Sex	-0.966 (0.381)	-1.231 (0.292)	-0.703 (0.495)	categorical (0=female, 1=male)
Twin	-0.569 (0.566)	-0.921 (0.398)	-0.215 (0.806)	categorical (0=singleton, 1=twin)
Group level/random effects (standard deviation)				
Birth Year	2.217 (9.183)	1.666 (5.289)	2.957 (19.246)	n = 30
Mother ID	0.766 (2.15)	0.493 (1.636)	1.025 (2.787)	n = 819

Supplementary Table 2: Bayesian GLMM estimates for first-year survival. The sample size was n = 2294. The table shows the posterior mean and 95% credible intervals and information on how each variable was encoded and transformed prior to modelling.

Term	Post.Mean	CI (2.5%)	CI (97.5%)	Info
Intercept	12.843	12.629	13.058	
Residual	1.264	1.22	1.309	
Population level/fixed effects				
SEL05 (1 copy)	0.166	0.043	0.289	categorical
SEL05 (2 copies)	0.212	-0.042	0.466	categorical
SEL07 (1 copy)	0.035	-0.1	0.169	categorical
SEL07 (2 copies)	0.237	-0.159	0.631	categorical
SEL18 (1 copy)	0.002	-0.117	0.123	categorical
SEL18 (2 copies)	-0.027	-0.276	0.223	categorical
F_{ROH}	-0.048	-0.108	0.011	z-transformed (x-mean(x))/sd(x)
Hindleg length	2.234	2.167	2.301	z-transformed (x-mean(x))/sd(x)
Sex	0.608	0.492	0.726	categorical (0=female, 1=male)
Twin	-0.446	-0.611	-0.276	categorical (0=singleton, 1=twin)
Group level/random effects (standard deviation)				
Birth Year	0.477	0.346	0.659	n = 30
Mother ID	0.66	0.58	0.742	n = 819

Supplementary Table 3: Bayesian GLMM estimates for August body weight in lambs. The sample size was n = 2286. The table shows the posterior mean and 95% credible intervals and information on how each variable was encoded and transformed prior to modelling.

Supplementary Methods

Single locus expectations for frequency declines.

For SEL07 and SEL18, we compared the empirical frequency changes over time to expectations based on a single-locus population genetic model for a large, random mating population. For simplicity, we assumed that the fitness effect associated with a given haplotype is caused by a single allele at a locus within the haplotype. We further assumed that the allele is fully recessive and that its selection coefficient *s* is equal to the degree of lethality associated with a haplotype. Specifically, we assumed the selection coefficients *s* for SEL07 and SEL18 to be -0.47 and -0.31, as we found 47% and 31% fewer homozygotes than expected for these two haplotypes, respectively. We set their initial frequencies *q* to 19% and 31% for SEL07 and SEL18, respectively, which correspond to the haplotype frequencies in the Soay sheep birth cohort of 1990. We then calculated the change of allele frequency Δq for the putative lethal allele per generation as (Table 2.2 in Falconer & Mackay, 1996):

$$\Delta q = -\frac{s \cdot q^2 \cdot (1-q)}{1 - s \cdot q^2}$$

Our empirical haplotype frequencies were recorded over 18 years from 1990 to 2018. To get an expectation for the frequency change based on the above single-locus model, we assumed a Soay sheep generation time of three years, and hence ran six iterations (assuming six generations) of the formula to get to the expected allele frequency in 2018.

Single locus expectations for equilibrium frequency under overdominance.

Haplotype SEL05 appears to be both semi-lethal when homozygote but also to potentially exhibit overdominance. Moreover, rather than declining, we found it's frequency to be relatively stable in the Soay sheep population, changing only from 20 to 23% in the years from 1990 to 2018. Therefore, we compared the observed frequency of the haplotype to the equilibrium frequency expected from a single locus model, with the same assumptions as above. Here, we used the equation for the

change in the allele frequency Δq of an allele given overdominance (Table 2.2 in Falconer & Mackay, 1996):

$$\Delta q = \frac{pq(s_1 p - s_2 q)}{1 - s_1 p^2 - s_2 q^2}$$

Using the same reasoning as above, SEL05's selection coefficient was set -0.27. However, in addition, we found that SEL05 heterozygotes are 6.6% more likely to survive each year. Therefore, to calculate an equilibrium frequency, we set the heterozygote fitness to 1, and *s1* (the non-deleterious homozygote) to -0.06, and *s2* (the deleterious homozygote) to -0.33. We then set $\Delta q = 0$ and solved the equation for q to get the equilibrium frequency of a deleterious allele in a single locus model with the same selection patterns as SEL05.

Literature

Stoffel, M. A., Johnston, S. E., Pilkington, J. G., & Pemberton, J. M. (2021). Genetic architecture and lifetime dynamics of inbreeding depression in a wild mammal. *Nature Communications*, *12*(1), 2972. doi: 10.1038/s41467-021-23222-9

Falconer, D. S., and Mackay, T. E. C., (1996). Introduction to Quantitative Genetics, 4th ed. Longman, Essex.
